# The Novel Perspectives of Adipokines on Brain Health

**DOI:** 10.3390/ijms20225638

**Published:** 2019-11-11

**Authors:** Thomas Ho-yin Lee, Kenneth King-yip Cheng, Ruby Lai-chong Hoo, Parco Ming-fai Siu, Suk-yu Yau

**Affiliations:** 1 Department of Rehabilitation Sciences, Faculty of Health and Social Sciences, The Hong Kong Polytechnic University, 11 Yuk Choi Road, Hung Hum, Hong Kong; thomas.hy.lee@connect.polyu.hk; 2 Department of Health Technology and Informatics, Faculty of Health and Social Sciences, The Hong Kong Polytechnic University, 11 Yuk Choi Road, Hung Hom, Hong Kong; 3 Department of Pharmacology and Pharmacy, Li Ka Shing Faculty of Medicine, The University of Hong Kong, 11 Sassoon Road, Pokfulam, Hong Kong; 4 Division of Kinesiology, School of Public Health, Li Ka Shing Faculty of Medicine, The University of Hong Kong, 5 Sassoon Road, Pokfulam, Hong Kong

**Keywords:** adipokine, adipose-brain axis, brain health, neurodegeneration, depression

## Abstract

First seen as a fat-storage tissue, the adipose tissue is considered as a critical player in the endocrine system. Precisely, adipose tissue can produce an array of bioactive factors, including cytokines, lipids, and extracellular vesicles, which target various systemic organ systems to regulate metabolism, homeostasis, and immune response. The global effects of adipokines on metabolic events are well defined, but their impacts on brain function and pathology remain poorly defined. Receptors of adipokines are widely expressed in the brain. Mounting evidence has shown that leptin and adiponectin can cross the blood–brain barrier, while evidence for newly identified adipokines is limited. Significantly, adipocyte secretion is liable to nutritional and metabolic states, where defective circuitry, impaired neuroplasticity, and elevated neuroinflammation are symptomatic. Essentially, neurotrophic and anti-inflammatory properties of adipokines underlie their neuroprotective roles in neurodegenerative diseases. Besides, adipocyte-secreted lipids in the bloodstream can act endocrine on the distant organs. In this article, we have reviewed five adipokines (leptin, adiponectin, chemerin, apelin, visfatin) and two lipokines (palmitoleic acid and lysophosphatidic acid) on their roles involving in eating behavior, neurotrophic and neuroprotective factors in the brain. Understanding and regulating these adipokines can lead to novel therapeutic strategies to counteract metabolic associated eating disorders and neurodegenerative diseases, thus promote brain health.

## 1. Introduction

Adipose tissues are recognized as highly dynamic endocrine tissues exhibiting extensive physiological functions [1]. Adipose tissue is composed of mature adipocytes as well as the stromal vascular fraction, where adipose-derived stem cells, blood cells, fibroblasts, and nerves reside [2]. White and brown adipose tissues are the two fat tissues in mammals. The adipocytes in white and brown adipose tissues are morphologically and functionally distinct. Brown adipose tissues are accountable for thermogenesis by their abundant mitochondria and extensive vascularization [3,4], whereas white adipose tissues are preferably responsible for energy storage in the form of triglycerides. During fasting conditions, insulin inhibits lipolysis in white adipose tissues, where triglycerides are hydrolyzed into fatty acids and glycerol to generate energy [5]. In response to the nutritional states, adipose tissue plays an endocrine role by synthesizing and secreting bioactive compounds termed adipokines. Adipokine secretion is essential to energy and metabolic homeostasis [6]. Adipsin and leptin are the first adipokines identified [7,8]. Since then, many pivotal adipokines, such as adiponectin, resistin, and tumor necrosis factor α (TNF-α), have been extensively studied in metabolism and metabolic syndromes [9]. Novel adipokines, including chemerin, omentin, apelin, and adipocyte fatty acid-binding protein, have been identified by their metabolic functions resembling former adipokines and their alterations afflicted by metabolic diseases in humans [10].

The adipose-brain axis was first established with the discovery of leptin as an endocrine hormone targeting the hypothalamus to regulate energy balance, satiety, metabolism, and body weight [11]. It was later demonstrated that leptin signaling is also involved in hippocampal neuroplasticity [12]. De la Monte et al. have suggested that energy metabolism, glucose utilization, and insulin sensitization are compromised in Alzheimer’s disease (AD) patients, whereas these pathological symptoms may contribute to AD neuropathology [13,14,15,16]. Since AD neuropathology highly resembles the pathology of diabetes mellitus, AD is now recognized as the ‘type 3 diabetes’. Adiponectin promotes insulin sensitivity [17], whereas adiponectin deficiency exacerbates insulin resistance [18] and AD neuropathology [19]. Concerns are raised regarding how metabolic syndromes are linked to neurodegenerative diseases and mood disorders [20,21]. Given that adipose tissue is a major organ to regulate metabolism through adipokines, understanding the central function of adipokines in the brain can illustrate how the adipose-brain crosstalk works to prevent metabolic diseases and to promote brain health.

Metabolic dysfunction could share common molecular mechanisms with neurodegenerative diseases [22]. Therefore, emerging studies aim at investigating how adipose tissue exerts neuroprotective effects and influences neuroplasticity through secreting adipokines. Notably, several adipokines can cross the blood–brain barrier and act on the brain directly [1], while a damaged barrier will hinder adipose-secreting compounds entering the brain [23]. At present, it is noticeable that adipokines can modulate neuroplasticity. Accumulated studies have shown that leptin and adiponectin pathways can regulate cell proliferation [24,25], survival [19,26,27], and synaptic plasticity [12,28] by modulating cellular metabolism [29,30] and suppressing inflammatory response [31,32,33] in the brain. Previous studies focus on the acute action of leptin on synaptic plasticity, whereas recent studies report insulin resistance afflicts circulating leptin levels and affects central neuroplasticity and cognitive functions. Besides, animal studies have demonstrated a possible link between metabolic disorders with aberrant circulating adipokine levels and cognitive behavioral deficits [34,35,36,37,38] or neuropathology [39,40,41].

This review summarizes the emerging evidence on the roles of five adipokines, including leptin, adiponectin, chemerin, apelin, and visfatin, on promoting neuroplasticity. Furthermore, this review will discuss how diet-, obesity-, and diabetes-related conditions alter the adipokine levels and impair neuroplasticity and cognitive function in terms of mood as well as learning and memory.

## 2. Effects of Conventional and Novel Adipokines on the Brain

The neuroendocrine roles of leptin and adiponectin have been widely studied. Leptin and adiponectin receptors are widely expressed across many brain regions, in particular, the hippocampus, a critical region for spatial learning and memory formation, and emotional regulation [42]. Both leptin and adiponectin are shown to affect synaptic plasticity in the hippocampus. However, the neuroprotective effects of other adipokines, including chemerin, apelin, and visfatin, are mostly unknown.

### 2.1. Leptin

Leptin is mostly secreted by white adipose tissue to regulate energy homeostasis by suppressing food intake and thereby reducing weight loss [43]. Circulating leptin levels are positively correlated with adiposity [44] and caloric intake [45,46,47,48,49]. In the obese population, increased adiposity is the primary cause of hyperleptinemia, a condition with high circulating leptin levels [44]. Obese subjects are refractory to the anorexigenic effect of leptin due to leptin resistance in the brain and peripheral tissues [50]. Similarly, patients with type 2 diabetes mellitus comorbid with obesity show both insulin-resistance and hyperleptinemia [51]. Multiple studies have demonstrated the effectiveness of exogenous leptin administration on improving insulin resistance in different obese and diabetic rodent models. Mouse models that feature obesity, hyperglycemia, hyperinsulinemia, particularly *db*/*db* (leptin-deficient) mice, and Zucker *fa*/*fa* (loss-of-function leptin receptor) rats resemble type 2 diabetic conditions in humans. Infusion of recombinant leptin for seven days is sufficient to ameliorate hyperglycemia and hyperinsulinemia in leptin-deficient *ob*/*ob* mice [52]. Leptin treatment can also improve insulin resistance, and glucose and lipid imbalances in some type 2 diabetic models [53,54,55,56]. However, mice receiving chronic (>20 weeks) high-fat diet are resistant to leptin even when leptin is directly infused into the cerebral ventricle of the brain [57,58,59,60]. Prominently, leptin also fails to improve diabetes and insulin resistance in type 2 diabetic patients comorbid with obesity [61,62]. These suggest that leptin replacement therapy is feasible when deficient. However, further elevation in the leptin-resistant state will not improve metabolic syndrome.

Adiposity hinges on excessive caloric intake and accumulation, whereas food consumption is a complex behavior integrating energy homeostasis, reward system, and stress. Leptin is an appetite hormone, where leptin receptors are expressed in multiple brain regions [63]. Leptin exerts dual actions to regulate anorexigenic-mediated energy homeostasis [64] as well as to suppress the food-cued reward circuit in the hypothalamus [65,66]. Hypothalamus is the main target of leptin in the brain by eliciting a homeostatic response to energy accumulation in accord with nutritional states. In response to energy accumulation, leptin inhibits orexigenic neurons expressing neuropeptide Y (NPY) and agouti-related peptide (AgRP) as well as activates the anorexigenic proopiomelanocortin (POMC) neurons in the hypothalamic arcuate nucleus (ARC). The inclusive anorexigenic effect prevails during energy accumulation after feeding together with an increased body fat oxidation, and hence suppresses leptin synthesis and secretion in a negative feedback loop [67]. On the other hand, plasma leptin levels are decreased by fasting before fat depletion [68], which in turn, disinhibits orexigenic action and stimulates the appetite.

Hedonic behavior in response to food is mediated by the mesolimbic circuit involving endocannabinoid [69], orexinergic [70], and dopaminergic signaling. The food-induced hedonic circuit can possibly override homeostatic feeding. The lateral hypothalamic (LH) together with the mesolimbic reward circuit, which involves the ventral tegmental area (VTA) and nucleus accumbens (NAc) predominate the hedonic circuit, where leptin elicits anorectic action by inhibiting multiple signaling along the circuit [71,72,73]. Orexin is a neuropeptide that mediates energy sensing, appetite, body weight, and reinforces reward behaviors [74,75,76]. Orexin-expressing neurons located in the LH innervate in the VTA, where leptin receptors are expressed [77]. Leptin can suppress activities of orexin-expressing neurons in the LH, as shown in electrophysiological recordings [77]. Besides, intra-LH leptin infusion abolishes high-fat diet-conditioned place preference mediated by orexin-expressing neurons [77]. Of note, endocannabinoid signaling is in part involved in the leptin inhibitory action on orexin-expressing neurons. Leptin deficiency and high-fat diet could enhance cannabinoid receptor-mediated presynaptic inhibitory control of orexin-expressing neurons in the hypothalamic ARC [69]. These findings taken together suggest the leptin suppresses hedonic behavior in the hypothalamus involving orexigenic and endocannabinoid signaling. In addition, dopamine release in the NAc is an adaptive response to palatable food consumption in association with reward learning and incentive salience [78,79,80]. A recent review has comprehensively addressed the crosstalk of leptin and dopamine signaling in inhibiting the food reward system through the LH-VTA-NAc neural circuit [81]. The LH GABA-ergic projection to the VTA expresses leptin receptors [82]. Leptin promotes GABA release from the LH to VTA is associated with reduced food consumption in leptin-deficient mice [82]. The inhibitory effect of leptin in the reward circuit is further supported by the lentiviral-mediated knockdown of the leptin receptor in the LH [83]. Finally, leptin receptors are also expressed in dopaminergic innervation from the VTA to the NAc. Intra-VTA infusion of leptin suppresses food intake [84]. Inhibition of dopaminergic neuron activity in the VTA neurons [85] and the reduced dopamine levels in the NAc [86] may downregulate the reward system. The underlying molecular signaling for leptin inhibition in the VTA dopaminergic neuron may activate the Janus kinase-signal transducer and activator of transcription proteins JAK/STAT/PI3K/mTOR pathway [84,87]. Significantly, long-term overexpression of leptin in the VTA causes leptin receptor desensitization [88]. Conversely, adenoviral-mediated knockdown of leptin receptor in the VTA promotes food intake and sucrose preference [89], and leptin antagonism or knockout in the VTA increases NAc dopamine levels [90]. The abovementioned studies concertedly suggest the overconsumption behavior and continuous body weight gain regardless of hyperleptinemia in obese subjects [91]. Enlarged adipocyte is the culprit of hyperleptinemia. And potentially, instead of exhibiting a stronger anorexigenic effect or a stronger inhibition of hedonic reward, prolonged hyperleptinemia may desensitize the leptin receptor-mediated signaling [60]. These result in a vicious cycle of hedonic eating and obesity.

Excessive caloric intake and energy accumulation are not the only causes of obesity. Chronic atypical antipsychotic medication is obesogenic in association with severe metabolic disorders, including hyperglycemia, insulin resistance, and hyperleptinemia [92,93,94,95,96,97,98]. In particular, weight gain, increased food consumption, and hyperleptinemia are frequently reported in psychotic patients receiving olanzapine medication [99,100]. Significant elevation of circulating leptin is detected in schizophrenic subjects receiving haloperidol medication (pre, typical antipsychotic treatment) versus risperidone and clozapine medication (post, atypical antipsychotic treatment) [101]. Elevated leptin levels and weight gain in schizophrenic patients treated with atypical antipsychotics may be involved in an adiposity-induced feedback mechanism [102]. Rodent studies further support the fact that the atypical antipsychotics, namely risperidone and olanzapine, are obesogenic as well as induce leptin expression and secretion [103,104,105,106]. Essentially, antipsychotics can act on multiple neuroendocrine pathways. Alongside with serotoninergic antagonism that may underline hedonic feeding behaviors and atypical weight gain [107,108,109], some atypical antipsychotics can contravene energy homeostasis by inhibiting peripheral adrenergic system [97]. β-3 antagonism by atypical antipsychotics can not only disrupt energy homeostasis [110,111] but also induce hyperleptinemia [112]. In sum, atypical antipsychotics are obesogenic by contravening brain anorexigenic and reward circuit as well as disrupting normal adipocyte functioning.

Besides, leptin receptors are found in the hippocampus and neocortex, suggesting their possible roles in the cognitive process [63]. Leptin resistance in obese and diabetic patients can be linked to impaired leptin receptor signaling [64] and leptin insensitivity at the blood–brain barrier [50,113]. Although both leptin mRNA and protein expressions are widely detected in the brain [114,115], circulating leptin can pass through the blood–brain barrier [116] and could function as a neurotrophic factor in modulating neuroplasticity and cognitive functions [117]. *db*/*db* mice, which show loss of function in leptin receptor signaling, display neuronal atrophy in the hippocampus, including reduced hippocampal progenitor cell proliferation [118], reduced dendritic branching and dendritic spine [119]. Besides, both *db*/*db* mice and Zucker *fa*/*fa* rats show spatial memory deficits in the Morris water maze task [118,120,121] and exhibit despair behaviors [122], suggesting that impaired leptin signaling could affect structural plasticity in the hippocampus. HFD-induced diabetic model [123] and *db*/*db* mice [124] display hyperleptinemia and leptin resistance. Enhancing leptin levels enhances neuroplasticity and cognitive function in rodents under physiological conditions. For example, intraperitoneal injection with 1 mg/kg recombinant leptin continuously for 14 days promotes hippocampal cell proliferation and survival of newborn neurons in healthy male mice [24]. An in vitro study has demonstrated that leptin promotes dendritic branching and spine maturation in cultured primary neurons [125]. Moreover, leptin elicits a rapid antidepressant effect by reducing depression-like behavior in the forced swim test and tail suspension test [126]. Intrahippocampal infusion with leptin immediately after training improves memory processing in T-maze footshock avoidance and step down passive avoidance tests [127], suggesting that leptin has a direct effect on influencing hippocampal function and enhances behavioral outcome. Notably, previous studies have shown that leptin treatment via systemic injection or intra-hippocampus injection to the dorsal part can improve spatial and context-cued learning and memory. Nonetheless, a study has reported that leptin infusion in the ventral hippocampus, but not in the dorsal hippocampus, impairs memory consolidation for the spatial location of food [128]. A recent review suggests that activating the leptin signaling pathway, specifically in the ventral hippocampus, could suppress spatial working memory [129]. Mechanisms of how leptin affects structural plasticity in the hippocampus are still unclear. Leptin receptors are coupled to JAK/STAT and PI3K/Akt signaling cascades in hippocampal neurons. Activation of the above cascades increases the production of manganese superoxide dismutase (Mn-SOD), an antioxidant enzyme, as well as the anti-apoptotic protein Bcl-xL. The synergistic antioxidant and anti-apoptotic effects may stabilize the mitochondrial membrane potential, and hence lessen mitochondrial oxidative stress to promote neuronal proliferation in the hippocampus [26]. Leptin treatment could increase hippocampal cell proliferation and survival by increasing mitochondrial functioning. In addition, leptin can also promote synaptogenesis via increasing expression of microRNA-132 (miR-132) [119]. Leptin induces long-term potentiation (LTP) by modulating post-synaptic signaling pathway and glutamatergic *N*-methyl-D-aspartate (NMDA) receptor. LTP is considered as a cellular mechanism of learning and memory formation. The NMDA receptor-dependent LTP in the CA1 region of the hippocampus underlies spatial memory formation [130]. Both *db*/*db* mice and Zucker *fa*/*fa* rats with defective leptin receptors impair hippocampal LTP formation [120], whereas infusion of 1 µM leptin into the hippocampal dentate gyrus enhances LTP [131]. Ex vivo studies also show that leptin can increase pharmacologically-isolated NMDA receptor-mediated excitatory postsynaptic currents (EPSCs) in the CA1 region in acute rat hippocampal slices [132] (Table 1).

Neuroprotective effects of leptin have also been shown in animal models. Eight weeks of systemic infusion with leptin reduces amyloid-β levels in the brain and serum of six-month old CRND8 transgenic mouse model of AD. Leptin treatment also improves object recognition and contextual fear learning [133]. Moreover, intracerebroventricular injection of leptin (1 µg) for ten days improves spatial memory in Y-maze and water maze tasks, as well as restores LTP in the CA1 region in an Aβ-induced AD rat model [134]. The neuroprotective effects of leptin could be linked to a leptin/JAK2/STAT3 signaling pathway [135] or leptin mediated PI3K/Akt/mTOR signaling pathway [136]. In sum, leptin can directly modulate structural and synaptic plasticity, however prolonged elevation in leptin levels could be detrimental to neuroplasticity under hyperleptinemia condition.

### 2.2. Adiponectin

Adiponectin is secreted by white adipose tissue and exerts anti-inflammatory effects on both endocrine and cardiovascular systems [137,138]. Adiponectin receptors (AdipoRs) express differentially in peripheral tissues. AdipoR1 is highly expressed in skeletal muscle, while AdipoR2 is abundant in the liver [139]. Hypoadiponectinemia, a condition of low circulating adiponectin, is commonly observed in obese and type 2 diabetic patients [140,141,142]. In line with clinical evidence, diabetic and obese mice also have reduced adiponectin expression in adipose tissues as well as the blood [143,144,145,146]. Circulating adiponectin levels are reduced in obesity [140,141,142], whereas the expressions of adiponectin receptors are increased, as evidenced by higher mRNA expressions of AdipoR1 in skeletal muscle in obese subjects [147] and AdipoR2 in insulin-resistant subjects [148]. Recombinant adiponectin treatment can reduce body weight and increase hepatic and muscular insulin sensitivity in an adiponectin-deficient mouse model [149]. Similarly, patients receiving rosiglitazone, an insulin sensitizer targeting the adipose tissue, experience increased plasma adiponectin by approximately two-fold [150]. These findings indicate that upregulating adiponectin expression can improve insulin sensitivity. Adiponectin receptors (AdipoR) are widely expressed in rodent brains, including the hypothalamus, brainstem, prefrontal cortex, and hippocampus, suggesting its role in the CNS in addition to its role in metabolism [27,139,151,152,153,154,155,156,157,158,159]. Overnight fasting by food deprivation for sixteen hours concomitantly downregulates mRNA expression of adiponectin and upregulates mRNA expression levels of AdipoR1 and APPL1 in the hypothalamic ARC [160], a brain area in the mediobasal hypothalamus regulating energy and glucose homeostasis [161].

Adiponectin could enter the blood–brain barrier since the low-molecular weight trimeric form of adiponectin is detectable in cerebrospinal fluid after intravenous injection in adiponectin-deficient mice [25,162]. Prominently, adiponectin is involved in energy homeostasis and appetite in concordance with leptin. A recent investigation shows that adiponectin promotes anorexigenic POMC activity in the hypothalamic ARC [163] possibly through PI3-K signaling [164]. Adiponectin elicits an excitatory effect on POMC neurons in a leptin-receptor dependent manner [163], whereas leptin synergistically potentiates adiponectin excitatory effect on POMC neuronal activity [163]. Simultaneously, adiponectin inhibits the neighboring orexigenic NPY/AgRP neurons, which then disinhibit POMC activity [163]. Further studies show that adiponectin action on the hypothalamic POMC activity may occur in a glucose-dependent reciprocal manner. Adiponectin infusion in the cerebral ventricle exerts an orexigenic effect at high glucose levels but is anorexigenic at low glucose levels [165]. Moreover, adiponectin increases the anorexigenic POMC activity at low glucose levels (2.5–5 mM), mimicking the fasting and physiological states, but decreases the POMC activity at high glucose levels (10 mM), mimicking a fed state [165] in electrophysiological recordings. The state-dependent adiponectin mechanism of action may act differentially on different molecular pathways: Adiponectin inhibits POMC neurons at high glucose via AMPK signaling, while it activates POMC neurons at low glucose via PI3-K signaling [165]. In contrast to the glucose-dependent effect in POMC, adiponectin enhances the activity of inhibitory GABA-ergic NPY neurons in a glucose-independent fashion [166]. Altogether, at low glucose conditions, adiponectin suppresses orexigenic NPY neurons and activates anorexigenic POMC neurons to attenuate appetite and food intake under conditions of fasting or low blood glucose. On the other hand, at high glucose levels, feeding behavior is nullified [165] as adiponectin inhibits both anorexigenic and orexigenic activities. Since leptin resistance and hypoadiponectinemia are symptomatic in obese and type 2 diabetic subjects, disrupted interplay of leptin and adiponectin may collectively impair anorectic actions and thus promote caloric intake and energy accumulation in obese and diabetic conditions. Hypoadiponectinemia is often documented in patients receiving atypical antipsychotics medication, including olanzapine and clozapine, in association with increased adiposity and hyperleptinemia [68,167,168]. Specifically, clozapine medication leads to body weight gain, high triglyceride profile, and hypoadiponectinemia [169,170]. GABA-facilitated orexigenic NPY activation and the subsequent suppression of anorexigenic POMC activity is suggested to be a potential mechanism of olanzapine-induced obesity [171]. The disrupted hypothalamic control of energy homeostasis may, therefore, lead to hyperleptinemia and hypoadiponectinemia. Still, some atypical antipsychotics, including risperidone and quetiapine, do not affect adiponectin levels [168,172,173,174].

AdipoR1 is expressed in dopaminergic neurons in the VTA [175]. The adiponectin receptor expression implicates the crosstalk of adiponectin and dopamine signaling pathways. Hedonic behaviors and affective behaviors are intertwined by dopamine signaling [176,177,178,179]. Recent research dissecting the role of adiponectin receptor in mediating anxiety reveals that intra-VTA infusion of adiponectin or the adiponectin receptor agonist AdipoRon suppresses dopaminergic neuron firing in an AdipoR1-dependent manner [175]. However, impaired adiponectin signaling due to adiponectin haploinsufficiency or AdipoR1 ablation increases dopaminergic activity and anxiety behavior. Adiponectin action shares similarities with the inhibitory action of leptin on the VTA dopaminergic neurons. Importantly, anxiety symptoms are often comorbid with metabolic disorders [180], where hypoadiponectinemia is indicative in obese and type 2 diabetic subjects [140,141,181]. It is speculated that the interplay of leptin and adiponectin may also exist in the VTA dopaminergic neurons to modulate reward and affective behaviors.

Patients with type 2 diabetes or dementia display decreased adiponectin levels [182,183,184]. High fat diet-induced and streptozotocin-induced diabetic conditions reduce AdipoR1 and AdipoR2 expressions in the hippocampus [27]. Adiponectin knockout reduces proliferating cells, differentiating cells, and cell survival in the hippocampus [185]. Adiponectin stimulates the proliferation of hippocampal progenitors through p38MAPK/GSK-3β/β-catenin cascade [157]. Moreover, adiponectin deficiency reduces the dendritic length, branching, and spine density of dentate granule neurons, particularly in early-born granule neurons in the hippocampal dentate gyrus [185]. Low levels of adiponectin are associated with cognitive dysfunction [184]. Chronic adiponectin deficiency impairs spatial memory and learning and induces anxiety-like behavior [19]. Moreover, AdipoR1 knockdown causes metabolic dysfunction and neurodegeneration in an association with the cognitive deficit in Morris water maze [186]. Overexpression of adiponectin in the brain [25] or infusion of recombinant adiponectin into the cerebral ventricle induces antidepressant effects [158,187,188]. The antidepressant effect could be linked to the activation of the AdipoR1/APPL1/AMPK pathway [25]. Rosiglitazone, a blood–brain barrier-impermeable PPARγ-selective agonist, enhances adiponectin levels and elicits antidepressant- and anxiolytic-like effects in wild-type mice, but not adiponectin-deficient mice or mice pretreated with PPARγ-selective antagonist [189].

The pro-cognitive effect of adiponectin in AD could be linked to its anti-inflammatory effect and enhanced effects on synaptic plasticity. Globular adiponectin predominantly binds to AdipoR1 and inhibits nuclear factor-κB (NF-κB) activation in macrophages, which in turn, suppresses its production of pro-inflammatory cytokines [190,191]. Therefore, adiponectin may reduce the extracellular deposition of amyloid-β via its suppressive effect on microglial activation. Moreover, adiponectin attenuates streptozotocin-induced Tau hyperphosphorylation and cognitive deficits through PI3K/Akt/GSK-3β pathway [192]. Activating AMPK can suppress GSK-3β action on Tau phosphorylation, whereas chronic adiponectin deficiency fails to phosphorylate AMPK, which in turn, enables GSK3β action on promoting Tau hyperphosphorylation, and subsequently results in AD-like neuropathology [19]. Nine-month-old adiponectin-deficient, 5×FAD mice show microglia activation together with elevated TNFα and IL-1β levels in the cortex and hippocampus [193]. In accordance with the previous study, an in vivo study illustrates that globular adiponectin exerts anti-inflammatory effects on microglia by reducing IL-1β, IL-6, and TNFα [33]. Pre-treatment with adiponectin can diminish TNFα and IL-1β release in vitro [193]. Adiponectin potentially exerts anti-inflammatory action on microglia via AdipoR1/NF-κB signaling [33,193]. Other studies have further demonstrated that adiponectin exhibits anti-inflammatory response dependent on PPARγ [194] or IL-4/STAT6 signaling pathway [191]. Recently, an ex vivo study has demonstrated that adiponectin exhibits a beneficial effect on hippocampal synaptic plasticity in 5×FAD mice. Acute perfusion for 10-min or 2-h incubation of 2.7 nM adiponectin can induce LTP in the hippocampal CA1 region in five to six months old 5×FAD mice. Acute incubation of adiponectin also increases expressions of AdipoR1 and AdipoR2, increases protein expressions of GluA1, GluN1, GluN2B, and PSD-95 as well as elicits anti-apoptotic and anti-inflammatory effects [195]. These findings suggest that adiponectin inhibits neuronal apoptosis and inflammatory mechanisms and promotes hippocampal long-term potentiation. Taken together, these data have suggested the neuroprotective and neurogenic effects of adiponectin in the hippocampus (Table 1).

### 2.3. Chemerin

Chemerin is a novel adipokine that acts autocrine, paracrine, and endocrine on different tissues [196] by binding to either chemokine-like receptor 1 [197], chemokine receptor-like receptor 2 [198] or G protein-coupled receptor 1 [199]. Chemerin is highly expressed in white adipose tissue [200] and acts as a pro-inflammatory cytokine [201] and anti-inflammatory cytokine. It promotes adipogenesis [200], angiogenesis [202], and inflammation [203]. Clinical study has shown that change of chemerin levels is linked to obesity because the circulating levels of chemerin is positively correlated with high body-mass index and elevated obesity-related biomarkers [204,205,206]. Similarly, high fat diet-induced obese mice display an increase in plasma chemerin levels, and this increase can be reduced by overnight fasting [207]. Another study has shown that chemerin administration exacerbates the glucose intolerance in obese and diabetic mice by reducing hepatic glucose uptake [208]. Chemerin deficiency results in insulin resistance in adipose tissue and liver, leading to elevated hepatic glucose production and increased blood glucose levels [209]. On the other hand, chemokine-like receptor 1 knockout mice have reduced glucose uptake in adipose tissue and skeletal muscle [210], whereas high-fat diet further exacerbates glucose intolerance, hyperinsulinemia and enhances insulin resistance in these mice [211].

Activation of the chemerin signaling pathway plays a vital role in the neuroendocrine axis by regulating appetite. Chemerin receptors are highly expressed in multiple rodent brain regions, including the prefrontal cortex, hippocampus, and hypothalamus [212]. Continuous intracerebral infusion of chemerin into photoperiodic-sensitive F344 rats can promote food intake [213], whereas chemokine-like receptor 1 deficiency reduces food intake and body weight [210]. Chemerin can also aggravate glucose intolerance [208]. Consistently, clinical data indicate that circulating chemerin levels are increased in patients with obesity, diabetes, and cardiovascular diseases [214].

A recent study has shown that intranasal administration of human recombinant chemerin (rh-chemerin) exerts neuroprotective effects in a rat model of neonatal hypoxia-ischemia brain injury [215]. Intranasal treatment with chemerin significantly reduces infarct volume and attenuates developmental delay 24 h after hypoxic-ischemic encephalopathy. Chemerin treatment significantly improves cognitive and sensorimotor performance in animals with hypoxic-ischemic encephalopathy. Moreover, recombinant chemerin treatment significantly reduces apoptosis and the expressions of pro-apoptotic markers [215], suggesting a neuroprotective role of chemerin. Chemerin signaling is proposed to mediate neuro-inflammatory action in the brain. It is reported that the expression of chemokine like receptor 1 (CMKLR1) is upregulated in AD patients, suggesting the role of central chemerin signaling in the progression of AD [216]. Systemic lipopolysaccharide administration is known to upregulate CMKLR1 expression and promote neuroinflammation [216]. This study has reported that CMKLR1 and Aβ42 are colocalized in hippocampal neurons of APP/PS1 AD mice, and CMKLR1 is involved in Aβ processing and clearance. CMKLR1 is detected in the hippocampus and prefrontal cortex, two critical brain regions involved in the pathology of depression [217]. Intracerebral administration of chemerin elicits antidepressant effects [218]. In the lipopolysaccharide-induced depression mouse model, intracerebral administrations of chemerin receptor agonist: Eicosapentaenoic acid-derived resolvins E1 (1 ng) or E2 (10 ng) produce antidepressant effects depending on ChemR23/mTORC1 pathway in the medial prefrontal cortex and hippocampal dentate gyrus [218] (Table 1), suggesting activating chemerin signaling could be a novel antidepressant target.

### 2.4. Apelin

Apelin, a peptide hormone secreted by adipocytes, is involved in insulin secretion [219], glucose and lipid metabolism [220], angiogenesis [221], blood pressure regulation [222], and food intake [223]. Apelin has similar properties as physical exercise does in metabolism, and so given entitled it as an exerkine [224]. Apelin can be proteolytically cleaved into several bioactive forms, including apelin-13, -17, and -36, as well as the pyroglutaminated apelin-13 isoform [225]. Apelin receptors are widely distributed in rodent brain and systemic tissues [226].

Apelin is necessary for balancing the fat composition and promoting insulin sensitivity. Apelin-knockout mice exhibit obese and diabetic symptoms, including increased abdominal fat mass, glucose intolerance, hyperinsulinemia, and hypo-adiponectinemia [227]. High-fat diet and high-sucrose consumption aggravate glucose and insulin intolerance in apelin-deficient mice [228]. Clinical study has reported a positive correlation between plasma insulin levels and apelin expression in adipocytes from obese subjects. Given this insulin-sensitizing property, apelin has become a promising therapeutic target for treating obesity and diabetes. Single intravenous injection of 200 pmol/kg Pyr(1)-apelin-13 can improve glucose intolerance in high-fat diet-induced obese and insulin-resistant mice [229]. Chronic apelin treatment (two to four weeks) improves insulin sensitivity, lowers blood glucose levels, and protect the animals from hyperinsulinemia and glucose intolerance in mice with obesity or insulin resistance [228,230]. Apelin treatment can also significantly reduce adiposity and plasma triglycerides [230]. Apelin is involved in lipid metabolism by inhibiting isoproterenol-induced lipolysis in cultured adipocytes [227]. Moreover, exogenous apelin reduces the number of differentiated adipocytes and increases the size of lipid droplets inside the cells, suggesting that apelin might suppress lipolysis [231]. In vitro studies reveal that apelin-13 and [Pyr1]-apelin-13 improve glycemia in an AMPK/eNOS-dependent [229] and PI3K/Akt-dependent [232] pathways respectively. These findings have collectively suggested that apelin treatment could be useful in reducing adiposity, improving insulin sensitivity, and improving diabetic conditions.

Similar to other adipokines, apelin exhibits anti-inflammatory properties [232] and possesses neuroprotective effects [233]. In an ischemia/reperfusion (I/R) stroke rat model, the post-stroke intracerebral injection of apelin-13 can significantly reduce the infarct volume [234]. Similarly, intracerebral infusion of apelin-13 significantly decreases blood–brain barrier permeability and increases vascular endothelial growth factor levels in post-stroke mice [235]. Furthermore, post-stroke treatment with intranasal apelin-13 for three days can reduce infarct volume, reduce neuronal apoptosis, suppress inflammation, and increase angiogenesis [236]. In mice with traumatic brain injury, intracerebral administration of apelin-13 reduces brain damage via suppressing autophagy [237]. Apelin-13 also elicits an anti-inflammatory effect by inhibiting microglia and astrocyte activation and upregulates hippocampal BDNF and TrkB expression in streptozotocin-induced sporadic AD rats. Furthermore, the apelin-13 treatment also promotes learning and memory performance, as evidenced by its effect on improving novel object recognition and spatial recognition through the BDNF/TrkB pathway [238]. In a rat model of depression, apelin-13 treatment elicits antidepressant effect and improves recognition memory via activating phosphatidylinositol 3-kinases (PI3K) and extracellular signal-regulated kinase 1/2 (ERK1/2) signaling pathways. The hippocampus is thought to be the critical brain region mediating the antidepressant-like response of apelin [239,240]. Similarly, intracerebral infusion of apelin-13 can ameliorate depression-like phenotypes in rats subjected to chronic restraint stress. Apelin-13 can also suppress hypothalamic-pituitary-adrenal axis hyperactivity, promote hippocampal BDNF expression, and restore hippocampal glucocorticoid receptor functions in rats with chronic stress [241] (Table 1).

### 2.5. Visfatin (Nicotinamide phosphoribosyltransferase, NAMPT)

Nicotinamide phosphoribosyltransferase (NAMPT) is an enzyme which catalyzes the biosynthesis of nicotinamide adenine dinucleotide (NAD^+^) in mammals [242]. There are two isoforms of NAMPT: Intracellular NAMPT (iNAMPT) and extracellular NAMPT (eNAMPT or visfatin) [242]. Growing evidence shows that NAD^+^/NADH metabolism in adipose tissue is linked to obesity and insulin resistance. In humans, visceral adipose NAMPT expression and serum NAMPT levels are positively correlated with obesity [243]. In mice, adipose NAMPT expression and NAD^+^ contents are markedly reduced by high-fat diet feeding [244,245], but increased by caloric restriction [246,247]. Adipocyte-specific *Nampt* knockout mice show severe insulin resistance in multiple organs under a chow-fed condition, which is independent of body weight and adiposity [248]. Loss of *Nampt* causes adipose tissue inflammation, increases plasma free fatty acid concentrations, and decreases adiponectin. Notably, oral administration with nicotinamide mononucleotide, a key NAD^+^ intermediate, can restore adipose tissue NAD^+^ biosynthesis and significantly restore insulin resistance, plasma adiponectin and free fatty acid concentrations in adipocyte-specific *Nampt* knockout mice. On the other hand, visfatin is secreted from white adipose tissue through sirtuin1 (SIRT1)-mediated deacetylation of iNAMPT [249]. At present, it remains uncertain whether visfatin can cross the blood–brain barrier. However, adipose-secreted visfatin serves as a neuroendocrine factor. Adipose tissue-specific *Nampt* knockout mice display a significant reduction in circulating visfatin, as well as reduced hypothalamic NAD^+^ content and SIRT1 activity [249]. On the other hand, adipose-specific *Nampt* knock-in mice show increases in hypothalamic NAD^+^ content, SIRT1 activity, and neural activity in response to fasting. Therefore, adipose-secreted visfatin has a strong implication on modulating brain functions.

Visfatin protects neurons against ischemia-induced injury because NAMPT overexpression can reduce infarct volume and improve long-term neurologic outcomes [250]. In a transient global cerebral ischemia model with 20-min carotid arteries occlusion, intracerebral infusion of 100 ng visfatin in the hippocampal CA3 region during cerebral reperfusion can reduce the caspase-3 activation and Bax/Bcl-2 ratio [251]. Visfatin can significantly reduce apoptosis and necrotic cell death in the CA1 region of the hippocampus, with improved memory deficits in I/R rats. The suppressed pro-apoptotic and enhanced anti-apoptotic mechanisms could contribute to the neuroprotective effects of visfatin [250]. In another study, transgenic mice overexpressing NAMPT globally increase NAD^+^ content and neuronal survival in the hippocampal dentate gyrus [252]. These mice also display better learning and memory performance in the water maze test upon middle cerebral artery occlusion. Post-ischemic intraperitoneal administration with nicotinamide mononucleotide for seven days can improve adult hippocampal neurogenesis [252]. Nicotinamide mononucleotide and NAD^+^ promotes proliferation and differentiation of neural stem cells in a NAMPT/NAD^+^/SIRT manner [252]. An in vitro study has concluded that cultured glia, but not neuron, can secrete visfatin under oxygen-glucose deprivation stress. Treatment of wild-type visfatin, but not H247A-mutant enzymatic-dead visfatin, can significantly attenuate oxygen-glucose deprivation-elicited cell death in both cultured mouse neuron and glia. Treatment of neutralizing antibody can, in turn, abolish the protective effect of extracellular visfatin on cell viability [253].

Although the neuroprotective benefits of visfatin towards neurodegenerative diseases require further investigation, growing evidence has indicated that NAD^+^ administration can improve cellular energetics and extend life span in rodents [254]. The evidence leads to the idea that the administration of NAD^+^ precursors, including nicotinamide mononucleotide and nicotinamide riboside, is a potential treatment to forestall disease progress in AD by improving brain energetics [255]. In the AD rat model with an intracerebral infusion with Aβ amyloid, intraperitoneal administration of 500 mg/kg nicotinamide mononucleotide improves learning and memory performance in water maze task [256]. Nicotinamide mononucleotide also attenuates Aβ oligomer-induced neuronal cell death, prevents long-term potentiation deficit, restores NAD^+^ and ATP levels, and eliminates reactive oxygen species in organotypic hippocampal slices [256]. Moreover, nicotinamide riboside treatment exhibits pro-cognitive function by increasing cortical NAD^+^ content and restoring long-term potentiation deficit in the hippocampal CA1 region of the Tg2576 AD mouse model. This study has suggested that treatment upregulating the NAMPT/NAD^+^ axis could prevent Aβ accumulation in the brain [257] (Table 1).

## 3. Novel Adipocyte-Derived Messengers on Brain Health

Lipokine refers to fatty acids that act like hormones and modulate lipid metabolism [258]. Notably, adipose tissue, which is one of the systemic sites with active lipid metabolism, secretes various lipids into the bloodstream to communicate with distant organs. Coincidentally, alterations in lipid metabolism may implicate the onset of brain diseases. For example, apolipoprotein E (ApoE) mediates systemic cholesterol metabolism and acts as a cholesterol carrier to neurons in the central nervous system. Human carriers of mutated apolipoprotein E allele ϵ4 are associated with an increased risk of AD [259]. Moreover, post-mortem studies have shown that AD patients display abnormal levels of ceramides, *n*-3 polyunsaturated fatty acids (PUFA), and PUFA-derived signaling lipids [260,261,262,263,264].

Lipokines, including monounsaturated palmitoleic acid and lysophosphatidic acid (LPA can exert endocrine effects on systemic tissues and have potential crosstalk with the central nervous system. Palmitoleic acid is a highly abundant fatty acid in the serum. Its circulating levels fluctuate dependent on metabolic states. Obese children and adults exhibit a higher plasma palmitoleic acid content compared to healthy control [265,266]. Besides, the change of palmitoleate could be associated with an increased risk of obesity, dyslipidemia, and insulin resistance [265,266,267,268]. On the other hand, lysophospholipid is a prominent class of lipid signaling molecules, whereas LPA is a crucial member. Circulating LPAs are synthesized by autotaxin, a phosphodiesterase produced by adipocytes [269]. Circulating autotaxin levels increase in obesity and insulin resistance [270,271,272,273,274,275,276], which is positively correlated with an increase in circulating lysophosphatidic acid [277,278]. A high-fat diet can perturb lipid composition in the brain. The altered lipid composition is evidenced by a surge of palmitoleic acid composition in the mouse brain after 14 days of high carbohydrate and high fatty acid diets [279]. Congruently, high-fat diet causes a significant elevation of LPA in mouse cortex after eight weeks of high-fat diet [280]. Importantly, over-nutritious food has detrimental effects on brain plasticity because brain diseases could be linked to dysregulated lipid metabolism [281,282,283,284,285,286]. The effects of palmitoleic acid and lysophosphatidic acid on brain health warrant further investigation.

### 3.1. Palmitoleic Acid (16:1n7)

Palmitic acid (16:0) is the most common saturated fatty acid in the human body. It can be consumed through diet or synthesized from other fatty acids, carbohydrates, and amino acids endogenously [287]. Under normal physiological conditions, palmitic acid accumulation is prevented by desaturation to palmitoleic acid (16:1n7) or by elongation to stearic acid (18:0), then further desaturation to oleic acid (18:1) [288,289]. Abnormal levels of palmitic acid have been documented in neurodegenerative diseases [290,291,292] that are related to dysregulated palmitic acid biosynthesis. Palmitoleic acid, a monounsaturated fatty acid, primarily originates from stearoyl-CoA desaturase 1-mediated de novo lipogenesis from palmitic acid in humans [287]. Palmitoleic acid is highly abundant in serum and adipose tissues [293]. *cis*-palmitoleate has been associated with increased insulin sensitivity and decreased lipid accumulation in the liver [258]. Various animal models have illustrated that *cis*-palmitoleate reduces the expressions of pro-inflammatory cytokines and adipokines in association with metabolic syndromes [258,294,295]. In vitro study also shows that palmitoleic acid increases lipolysis and lipase content in white adipose tissue in a PPARα-dependent manner [296]. Other than adipose tissues, palmitoleic acid can enhance whole-body glucose disposal [297] and improve circulating lipid profiles in both rodents and humans [298]. Therefore, palmitoleic acid is considered a lipokine.

Fatty acid-binding proteins (FABP) are lipid chaperones that regulate lipid trafficking and responses in cells [299,300]. In humans, circulating adipose-derived fatty acid-binding protein (A-FABP) levels are substantially higher than other adipokines, including leptin, resistin, TNF-α, and IL-6 [301,302,303]. Circulating A-FABP is markedly increased in both obese men and women [304], suggesting that A-FABP may involve in modulating insulin sensitivity and lipid metabolism in distant organs. Of note, A-FABP is also considered as an adipokine, which is secreted from adipocyte-derived exosomes by exocytosis [305]. Critically, deficiencies in A-FABP and epidermal fatty acid-binding protein (E-FABP) can activate SCD1 activity, leading to a subsequent elevation in palmitoleate concentration in adipose tissue [306,307]. A-FABP deficient mice exhibit improvement in systemic glucose, lipid metabolism and insulin resistance in association with diet-induced and genetically-disposed obesity [308]. The A-FABP and E-FABP double mutant mice are resistant to diet-induced obesity, insulinemia, type 2 diabetes, dyslipidemia, and fatty liver disease [309,310]. The dysregulated SCD1-mediated de novo synthesis of palmitoleic acid enhances whole-body glucose and fatty acid metabolism [311].

Although it requires more solid evidence to demonstrate the effect of adipocyte-secreted palmitoleic acid and A-FABP on brain health, it is reported that stearoyl-CoA desaturase activity and levels of palmitoleic acid in the brain are associated with AD [312]. Liquid chromatography-mass spectrometry analysis has revealed elevated levels of several monounsaturated fatty acids, including palmitoleic acid, in the mid-frontal cortex, temporal cortex, and hippocampus of AD patients as compared to their age-matched counterparts [312]. This increase is strongly associated with cognitive dysfunction and the increased expression of stearoyl-CoA desaturase in the brain [266,313,314,315,316,317]. Further studies have shown that AD patients have increased expressions of various stearoyl-CoA desaturase isoforms, including SCD-1 [312]. Coherently, stearoyl-CoA desaturase activity is negatively correlated to cognitive measures [312]. Levels of free palmitoleic acid and the desaturation index are decreased in frontal cortices in aged dogs treated with antioxidant, and vice versa [318]. The study implicates that altering stearoyl-CoA desaturase activity and reducing palmitoleic acid levels in the brain may improve cognitive performance [318].

### 3.2. Lysophosphatidic Acid

Neuroinflammation is commonly associated with type 2 diabetes [319], and obesity [320]. High-fat diet feeding results in profound central and peripheral inflammation [321,322]. Autotaxin is a lysophospholipase that synthesizes lysophosphatidic acid (LPA). Autotaxin is also regarded as an adipokine due to its role in promoting adipocyte differentiation [269]. Evidence suggests that the autotaxin-mediated LPA biosynthesis contributes to the majority of extracellular LPA, in which adipocyte-specific autotaxin contributes to most of the circulating LPA [269,272]. Further investigations suggest that circulating LPA and autotaxin levels are linked to adipocyte differentiation and obesity [269,271]. Autotaxin expression is upregulated in obese patients and obese *db*/*db* mice due to the accumulation of triglycerides in the adipocytes [271]. A recent study has reported that adipose-derived autotaxin serves as an inflammatory cytokine of diet-induced obesity [323].

LPA signaling mediates inflammation [324], angiogenesis [325], brain development [326], and neurogenesis [327] in the brain. Specifically, LPA exerts a pleiotropic effect on various brain cell types, including neurons, astrocytes, microglia, and oligodendrocytes that show LPA receptors expression [328]. LPA promotes neuronal differentiation in cortical neural progenitor cells through LPA1 receptor/G_i/o_ pathway [329], whereas axonal branching is induced in hippocampal cell cultures through LPA3 receptor/G_q_/Rho family GTPase 2 (Rnd2) pathway [330]. Mainly, LPA1 is essential in mediating synaptic plasticity and learning. In vitro studies have shown that overexpression of LPA1 increases dendritic spine density and size [331], potentially through protein kinase C (PKC)/Rho and PKC/Rac cascades [332,333]. It is speculated that LPA1 deficiency can cause schizophrenia-like behaviors, including impaired spatial memory retention and increased anxiety-like behavior [334,335,336]. Furthermore, the role of LPA and its receptors in anxiety-like behavior and learning has been confirmed by later studies using LPA1-null mice. These mice show normal survival but display aberrant hippocampal neurogenesis and decreased BDNF levels [327], as well as increased anxiety-like behavior and memory deficits [337]. LPA mediates astrocyte proliferation through the LPA1 receptor [338]. Interestingly, LPA promotes astrocyte-neuronal crosstalk through laminin-mediated EGFR/MAPK signaling to facilitate neuronal differentiation [339]. LPA triggers microglial migration through a Ca^2+^-dependent K^+^ channel [340,341], and reduces oxidative stress [342,343]. Moreover, LPA can promote the retraction of oligodendrocyte processes in mature oligodendrocytes [344] as well as promotes the formation of oligodendrocyte processes in differentiating oligodendrocytes [345].

In addition to a metabolic disorder, an altered systemic lipid metabolism may have a neurological impact [346] through autotaxin-LPA signaling. Clinical study has reported that mild cognitive impairment and AD patients have significantly higher levels of autotaxin in cerebrospinal fluid [347]. Furthermore, the autotaxin levels are positively correlated to neuroinflammatory markers, β-amyloid, and tau in the cerebrospinal fluid [347]. In vitro study has shown that LPA could increase Aβ production by upregulating the expression of β-secretase expressions in the N2a neuroblastoma cell line expressing wild-type presenilin 1 and Swedish mutant APP [348]. Potentially, LPA-induced BACE1 promoter activation is mediated by PKCδ/MEK/MAPK/p90RSk/CREB signaling cascades [348,349,350,351,352,353]. Moreover, LPA may be involved in tau hyperphosphorylation and paired helical filaments formation, which are the pathological patterns in AD brains through Gα_12/13_/RhoA/Rock/GSK-3β pathway [354,355], suggesting potential connection among LPA, microfilament dynamics, and AD pathogenesis.

## 4. Conclusions

**Figure 1:**
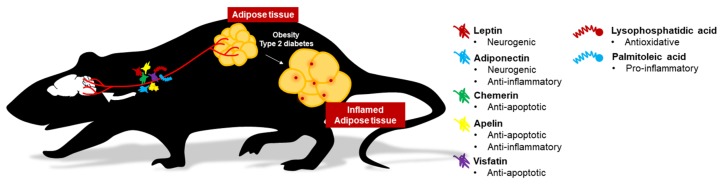
Potential mechanism of adipokine actions on brain health. Adiponectin and leptin can cross the blood–brain barrier to promote neuroplasticity. Other adipokines and lipokines show profound effects on mediating neurogenic and neuroinflammatory mechanisms.

It is an emerging research field to understand the functions of different adipokines in regulating eating behavior, hippocampal plasticity, and neuronal protection. This review has denoted five adipokines (leptin, adiponectin, chemerin, apelin, and visfatin) and two lipokines (palmitoleic acid and lysophosphatidic acid) (Figure 1). The review has summarized the currently available evidence for their novel function in the brain. First, it is evident that the aberrant adipokine production not only renders the susceptibility to metabolic syndromes in genetically deficient rodent models of various adipokines but also the vulnerability to neurodegeneration, especially in models of impaired leptin-signaling. Accumulated evidence has indicated that diet-induced obese or diabetic rodent models show abnormal levels of leptin, adiponectin, chemerin, apelin, and visfatin. Notably, circulating adiponectin and visfatin levels are reduced in diet-induced obesity, with associated impairments in hippocampal plasticity and cognitive behaviors [356]. Due to the multifaceted effect of adipokines on systemic and brain health, dysregulated adipokine secretion could be one of the causes that underlie the comorbidity of metabolic syndromes with neurodegenerative disorders [357,358]. Thus, all these investigations have highlighted the prominent roles of adipokines on brain health. Second, evidence also shows that leptin and adiponectin can elicit their action directly in the brain by crossing the blood–brain barrier. Leptin is not only a satiety regulator through anorexigenic and dopaminergic controls, but ample evidence also supports its role in promoting neuroplasticity in the hippocampus [12]. On the other hand, adiponectin is a chief metabolic regulator in glucose and fatty acid metabolism with insulin-sensitizing ability in both peripheral and central systems. Current findings have suggested the direct effect of adiponectin on synaptic plasticity [195,359] and microglial activation [193]. Both exogenous leptin and adiponectin can exert pro-cognitive effects in mouse models. Besides, leptin and adiponectin signaling pathways are defective in AD brains [360]. Hence, increasing leptin and adiponectin signaling can be a potential therapeutic target for AD [361,362]. Third, emerging literature has also revealed the central function of three novel adipokines: Chemerin, apelin, and visfatin. Inflammatory response and apoptosis are typical in neurodegenerative diseases [363,364] and ischaemic stroke [365,366]. These novel adipokines are potential therapeutic targets, which modulate anti-inflammatory and anti-apoptotic effects in the hippocampus. Finally, this review has highlighted lipokines are a lipid signaling molecule secreted by adipocytes with potential endocrine effect acting on the brain [367]. Still, the potential of lipokines is mostly unknown, future studies will be required to illustrate its properties and function in neuroplasticity and cognitive functions.

Overall, adipokines emerge as viable therapeutic targets towards neurodegenerative diseases. Adipokine-based therapeutic approaches could be future pharmaceuticals to obliterate neurodegenerative disorders. More in-depth investigations on the above-mentioned adipokines and its crosstalk with the brain can open up new therapeutic targets to treat neurodegenerative diseases.

## Figures and Tables

**Table 1:** Effects of adipokines on neuroplasticity, neuroprotection, and cognitive behaviors.

Animal Model		Treatment	Delivery Route	Neurological Effects	References	Year
**Leptin**						
C57BL/6J (healthy)	mice	14 days 1 mg/kg recombinant rat leptin	i.p.	Increased cell proliferation and survival in the hippocampal dentate gyrus	Garza et al. [24]	2008
C57BL/6JJ (healthy)	mice	30 min after injecting 1 mg/kg leptin	i.p.	Reduced depressive behavior in forced swim and tail suspension tests	Liu et al. [127]	2010
4 and 12 m.o. SAM-P8	mice	0.25–0.5 µg mouse recombinant leptin post-training	Intra-hippo	Improved memory retention in T-maze footshock avoidance and step-down inhibitory avoidance.	Farr et al. [118]	2006
Sprague-DawleyJ (healthy)	rats	1.0 µM leptin	Intra-DG	Enhanced LTP	Wayner et al. [131]	2004
6 m.o. TgCRND8 AD	mice	8 weeks 20 µg leptin daily	Systemic infusion by osmotic pump	Improved object recognition Improved fear-associated learning	Greco et al. [133]	2010
Aβ1-42-assaulted	rat	10 days 1 µg leptin daily	i.c.v.	Improved spatial memory in Y maze and water maze Enhanced LTP	Tong et al. [134]	2015
**Adiponectin**						
HFD-induced stress and naïve C57BL/6J	mice	0.1–0.3 µg globular adiponectin	i.c.v	Reduced depressive behavior	Liu et al. [158]	2012
C57BL/6J (healthy)	mice	Overexpression of adiponectin by adenovirus	i.c.v	Reduced depressive behavior in forced swim test	Yau et al. [25]	2014
Sedentary or environmentally enriched C57BL/6J	mice	1 mg/kg globular adiponectin	i.v.	Reduced depressive behavior in forced swim test	Nicolas et al. [187]	2015
Corticosterone-induced stress C57BL/6J	mice	0.3 µg globular adiponectin	i.c.v.	Reduced microglial response Reduced pro-inflammatory cytokine secretion from microglia	Chabry et al. [188]	2015
*Adipo*^+/+^ & ^+/-^ C57BL/6J mice	mice	10 mg/kg rosiglitazone 1 or 3 h before test	i.p.	Reduced depressive and anxiety-like behavior	Guo et al. [189]	2017
STZ-infused Sprague–Dawley	rats	0.1 µg/g adiponectin	i.c.v.	Improved learning and memory Increased hippocampal dendritic and spine complexities Reduced tau hyperphosphorylation	Xu et al. [192]	2018
**Chemerin**						
Hypoxic-ischemic encephalopathy in P10 Sprague-Dawley	rats	9 μg/kg rh-chemerin, 3 doses	i.n.	Better learning and memory functions Reduced apoptosis	Zhang et al. [215]	2019
LPS-induced depression BALB/c	mice	500 ng chemerin	i.c.v.	Reduced depressive behavior in forced swim and tail suspension tests	Deyama et al. [218]	2018
LPS-induced depression BALB/c	mice	Resolvin E1	i.c.v. (1 ng) and bilateral intra-mPFC and intra-DG (50 pg/side)	Reduced depressive behavior in forced swim and tail suspension tests	Deyama et al. [218]	2018
LPS-induced depression BALB/c	mice	10 ng resolvin E2	i.c.v.	Reduced depressive behavior in forced swim and tail suspension tests	Deyama et al. [218]	2018
**Apelin**						
Cerebral I/R Wistar	rats	50 ng/kg apelin-13 post-ischemia	i.c.v.	Reduced infarct volume	Xin et al. [234]	2015
Cerebral I/R *AQP4*^+/+^ & ^-/-^	mice	50 ng/kg apelin-13 post-ischemia	i.c.v.	Decreased BBB permeability	Chu et al. [235]	2017
Cerebral I/R C57BL/6J	mice	4 mg/kg, 3 doses for three days post-ischemia	i.n.	Reduced infarct Reduced neuronal apoptosis	Chen et al. [236]	2015
TBI CD-1	mice	50 µg apelin-13	i.c.v.	Suppressed autophagy	Bao et al. [237]	2015
STZ-infused AD Sprague–Dawley	rats	2 µg, daily for 4 weeks	i.c.v.	Improved object and spatial recognition Suppressed neuroinflammation	Luo et al. [238]	2019
Forced-swimming stressed Wistar	rats	2 µg, three doses in 24 h before FST	i.c.v.	Reduced hedonic deficit in learned helplessness Improve object recognition	Li et al. [238]	2016
Chronic water-immersion restraint stress Wistar	rats	2 µg, daily for 7 days	i.c.v.	Reduced depressive behavior in sucrose preference and tail suspension tests	Dai et al. [241]	2018
**Visfatin**						
Cerebral I/R	mice	pThy1:NAMPT	transgenic	Reduced infarct volume Reduced neurological deficits Increased striatal and corpus callosum myelination	Jing et al. [250]	2014
Cerebral I/R Wistar	rats	100 ng visfatin during reperfusion	Intra-CA	Suppressed apoptosis Enhanced anti-apoptotic mechanism	Erfani et al. [251]	2015
Cerebral I/R C57BL/6J	mice	pIRES2::NAMPT	transgenic	Improved learning and memory	Zhao et al. [252]	2015
Cerebral I/R C57BL/6J	mice	500 mg/kg NMN daily for 7 days	i.p.	Promoted hippocampal neurogenesis	Zhao et al. [252]	2015
Aβ-infused AD Sprague-Dawley	rats	500 mg/kg NMN daily for 10 days	i.p.	Improved learning and memory	Wang et al. [256]	2016
Tg2576 AD	mice	250 mg/kg nicotinamide riboside daily for 3 months	i.p.	Improved object recognition Increased cortical NAD^+^ Abolished long-term potentiation deficits	Gong et al. [257]	2013
